# Ocular Findings in Women with Cervical HPV Positivity and Cervical Dysplasia Undergoing Loop Electrosurgical Excision Procedure: A Prospective Cross-Sectional Study

**DOI:** 10.3390/medicina62050969

**Published:** 2026-05-15

**Authors:** Nazlı Aylin Vural, Burak Mergen, Serkan Güler, Cafer Yelkenci, Ecem Esma Yeğin, Nilüfer Çetinkaya Kocadal

**Affiliations:** 1 Department of Gynecologic Oncology, Yozgat State Hospital, Yozgat 66100, Turkey; 2 Molecular Oncology, Graduate School of Education, Istinye University, Istanbul 34396, Turkey; 3 Department of Gynecologic Oncology, Basaksehir Cam and Sakura State Hospital, Health Sciences University, Istanbul 34668, Turkey; 4 Care Vision Freiburg, 79098 Freiburg im Breisgau, Germany; 5 Department of Ophthalmology, Basaksehir Cam and Sakura City Hospital, Health Sciences University, Istanbul 34668, Turkey; 6 Department of Ophthalmology, Çorlu State Hospital, Tekirdağ 59850, Turkey; 7 Department of Obstetrics & Gynecology, Basaksehir Cam and Sakura State Hospital, Health Sciences University, Istanbul 34668, Turkey; 8 Biostatistics and Medical Informatics, Istinye University, Istanbul 34396, Turkey; 9 Gynecologic Oncology, Department of Obstetrics and Gynecology, Yeditepe University, Istanbul 34755, Turkey

**Keywords:** cervical dysplasia, human papillomavirus, conjunctival papilloma, ocular surface lesions, LEEP

## Abstract

*Background and Objectives*: To evaluate ocular findings in women with cervical HPV positivity undergoing loop electrosurgical excision procedure (LEEP) for cervical dysplasia and to assess conjunctival/eyelid papilloma and other ocular findings in this cohort. *Materials and Methods*: This single-center, prospective, cross-sectional observational study was conducted at a tertiary referral hospital between September 2021 and October 2023. Women in whom LEEP was planned because of cervical dysplasia were eligible for inclusion. Patients who consented to ophthalmologic evaluation underwent preoperative eye examination and were included in the study. For the final analysis, women with cervical HPV negativity were excluded. Demographic characteristics, cervical HPV status and subtype, colposcopic biopsy histopathology, final LEEP histopathology, and ophthalmologic findings were recorded prospectively. Ocular findings were classified as normal ocular findings, conjunctival/eyelid papilloma, or other ocular findings. *Results*: A total of 75 women were included in the final analysis. Normal ocular findings were observed in 66 women (88.0%), conjunctival/eyelid papilloma lesions were identified in 3/75 (4.0%), and other ocular findings were identified in six (8.0%). There were no significant differences among the ocular finding groups in age, gravida, or parity. Alcohol use differed across ocular finding groups (*p* = 0.039), although this finding was based on only three patients in the papilloma group and should be regarded as exploratory. Ocular findings were associated with colposcopic biopsy histopathology (*p* = 0.016) but not with final LEEP histopathology. In exploratory adjusted analyses, no independent predictor of conjunctival/eyelid papilloma or any other ocular pathology was identified. *Conclusions*: In this cohort, the majority of patients had normal ocular findings, and conjunctival/eyelid papilloma lesions were identified in only three patients. The observed ocular findings did not support a clinically meaningful cervical–ocular association in this cohort. Further studies with ocular tissue-based HPV testing are needed to investigate whether HPV is present in ocular lesions and whether these findings correspond to cervical HPV results.

## 1. Introduction

Human papillomavirus (HPV) has been identified as the primary etiologic agent in cervical cancer and cervical precancerous lesions [1]. Its well-established role in cervical carcinogenesis has led to the focus on HPV in current cervical screening, colposcopic assessment, and management of cervical dysplasia, as reflected in current guideline recommendations [2,3,4,5]. Despite advances in vaccination and screening programs, cervical cancer remains a pervasive global health issue, with an estimated 662,301 new cases and 348,874 deaths reported worldwide in 2022 [6].

However, HPV-related epithelial disease is not confined to the cervix [7,8]. Previous studies have demonstrated that HPV significantly contributes to malignant diseases across multiple mucosal sites, including the anus, vulva, vagina, penis, and head and neck region [7,9]. Among extra-cervical sites, the oropharynx has a well-established association with HPV-related malignant disease [10]. In the most recent systematic review and meta-analysis, pooled HPV positivity was 42% in oropharyngeal squamous cell carcinoma, and HPV16 comprised 89% of HPV-positive oropharyngeal tumors [10]. The incidence of HPV-related oropharyngeal cancer has increased substantially in developed countries [11].

This broader mucosal presentation has given rise to questions regarding the potential involvement of other exposed epithelial surfaces in HPV-related diseases [12,13,14]. While the ocular surface is one such site, the evidence is more limited and lesion-specific. The strongest association has been reported for conjunctival papilloma [15]. In the case series examining conjunctival papillomas and normal conjunctiva, HPV was detected in 86 of 106 papillomas (81%), predominantly low-risk HPV types 6 and 11, whereas all 20 normal conjunctival specimens were HPV-negative [16]. These findings support an association between HPV and conjunctival papilloma; however, they do not establish that the conjunctiva behaves as a uniform HPV-driven site [15,17].

The correlation between HPV and other ocular surface lesions is less consistent. Pterygium remains controversial [18], whereas the evidence is stronger for conjunctival intraepithelial neoplasia and conjunctival squamous cell carcinoma, with meta-analyses reporting pooled odds ratios of 8.42 and risk ratios of 3.13 for these associations [17,19]. More recent analyses also showed that HPV was 9.2 times more frequent in conjunctival cancers than in benign tissues [20]. Overall, the existing literature supports a potential role for HPV in selected ocular surface lesions, although this relationship has not been consistently observed across all lesion types [17,19,20].

Most published studies on ocular HPV are focused on pathology, lesions, or ophthalmology, and prospective data in gynecologic cohorts remain limited. In this study, we assessed ocular findings in women undergoing a loop electrosurgical excision procedure (LEEP) **for** cervical dysplasia who also consented to ophthalmologic examination. The primary aim was to determine the presence of synchronous conjunctival/eyelid papilloma, while our secondary aim was to describe other ocular findings identified on examination. Associations with cervical histopathology and HPV subtype were evaluated as secondary analyses.

## 2. Materials and Methods

### 2.1. Ethics Approval and Consent

The study was approved by the Local Ethics Committee of Başakşehir Çam and Sakura City Hospital, University of Health Sciences (approval number: 211; study protocol number: 2021-211; approval date: 22 September 2021) prior to patient enrollment. Written informed consent was obtained from all participants before inclusion in the study. The study was conducted in accordance with the principles of the Declaration of Helsinki. The manuscript was prepared in accordance with the Strengthening the Reporting of Observational Studies in Epidemiology (STROBE) statement.

### 2.2. Study Design and Setting

This single-center, prospective, cross-sectional, observational study was conducted at the Gynecologic Oncology Surgery Clinic of Başakşehir Çam and Sakura City Hospital, a tertiary referral hospital in Istanbul, Türkiye, between September 2021 and October 2023. Women in whom LEEP was planned for the presence of cervical dysplasia were considered eligible for the study. Among these patients, those who consented to ophthalmologic evaluation underwent preoperative eye examination and were included in the study cohort. For the final analysis, only women with cervical HPV positivity documented on testing performed at our institution were included.

### 2.3. Study Population and Variables

The study population comprised women who received treatment within the cervical dysplasia management of the gynecologic oncology clinic. As part of routine care, women referred with abnormal cervical cytology/histology and/or cervical HPV positivity underwent colposcopic evaluation, and cervical biopsy and/or endocervical curettage were performed as indicated. Patients with cervical dysplasia requiring excisional treatment proceeded to LEEP, and the final histopathologic findings of the excision specimen were recorded.

For each participant, demographic characteristics, reproductive history, cervical diagnostic findings, and ophthalmologic examination results were prospectively documented. Baseline demographic and clinical variables included age, gravidity, parity, age at first intercourse, education level, contraception use, smoking status, alcohol use, and chronic disease history. Variables related to cervical disease included cervical HPV status and subtype, colposcopic cervical biopsy findings, and the final histopathologic results of the LEEP specimen. For histopathologic analyses, cervical intraepithelial neoplasia grade 1 (CIN1) was defined as low-grade CIN, and CIN grades 2 and 3 (CIN2+) were defined as high-grade CIN. Invasive cervical cancer was analyzed independently when it was present in the final LEEP histopathology. Each patient underwent a slit-lamp biomicroscopic ocular surface examination prior to surgery. The outcomes were documented and subsequently utilized for further ocular assessment.

During the study period, 362 women underwent LEEP, 105 patients consented to participate and underwent ophthalmologic evaluation, and 30 were excluded from the final analysis because cervical HPV testing (Cobas^®^ 4800 HPV test (Roche Molecular Systems, Pleasanton, CA, USA)) performed at our institution was negative. The final analytical cohort consisted of 75 women.

### 2.4. Ophthalmologic Assessment

All participants who consented to ophthalmologic evaluation underwent slit-lamp biomicroscopic examination. Clinical assessment was based on biomicroscopic examination findings. The examinations were independently performed by two ophthalmologists (BM and SG) at separate time points, both blinded to the patients’ clinical and HPV status. Only cases with interobserver agreement were included in the final analysis. Ocular findings were systematically documented for each patient, and additional evaluations were performed when clinically indicated. Among the women who underwent ophthalmologic evaluation, both cervical HPV positive and HPV negative patients were present. For the final analysis, only women with cervical HPV positivity were included.

### 2.5. Outcome Definitions

Ocular findings were categorized into three groups: normal ocular findings, conjunctival/eyelid papilloma lesions, and other ocular findings. The category of other ocular findings included pterygium, pinguecula, and benign eyelid masses. The primary outcome was the frequency of conjunctival/eyelid papilloma lesions among the cohort. Secondary analyses assessed the distribution of ocular findings according to colposcopic biopsy results, final LEEP pathology, and HPV subtype. Additionally, comparisons were made between women with and without ocular pathology regarding age, gravidity, parity, and age at first sexual intercourse.

### 2.6. Statistical Analyses

Statistical analyses were conducted using RStudio software, version 2025.09.1+401 (R Foundation for Statistical Computing, Vienna, Austria). Patients were categorized into three groups based on ophthalmologic examination findings: normal ocular findings, conjunctival/eyelid papilloma lesions, and other ocular findings. The ocular finding category served as the sole grouping variable in the present analysis.

Continuous variables were summarized as mean ± standard deviation and as median with minimum–maximum values. Because the ocular finding groups were unequal in size and the papilloma group included a small number of patients, comparisons of continuous variables across the three groups were performed using the Kruskal–Wallis test. When the global test was statistically significant, pairwise post hoc comparisons were planned using the Wilcoxon rank-sum test with Benjamini–Hochberg adjustment.

Categorical variables were summarized as frequencies and percentages within each ocular finding group. Associations between categorical variables and ocular finding groups were assessed using Pearson’s chi-square test when its assumptions were met. When expected cell counts were small, Fisher’s exact test with Monte Carlo simulation was used. Variables with only one non-empty category were not considered testable. A two-sided *p* value less than 0.05 was considered statistically significant.

Because conjunctival/eyelid papilloma lesions and overall ocular pathology events were rare, exploratory Firth penalized logistic regression analyses were also performed. Two binary outcomes were evaluated: conjunctival/eyelid papilloma versus non-papilloma ocular findings, and any ocular pathology versus normal ocular findings. Any ocular pathology was defined as the presence of conjunctival/eyelid papilloma or other ocular findings. Given the limited number of outcome events, models were restricted to a small set of covariates. Results are reported as odds ratios with 95% confidence intervals and two-sided *p* values.

## 3. Results

### 3.1. Baseline Characteristics According to Ocular Finding Groups

**Figure 1:**
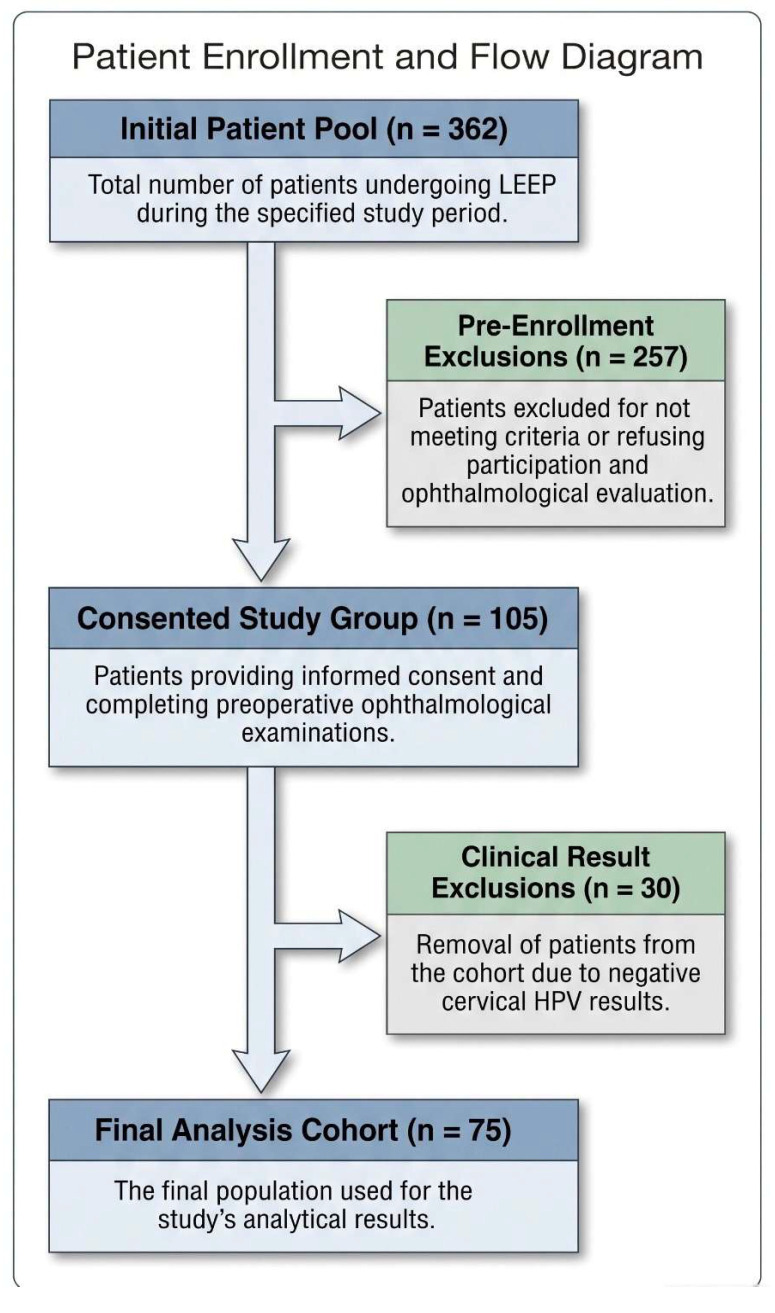
Flow diagram of patient selection and final analytical cohort.

A total of 75 women were included in the final analysis. During the study period, 362 women underwent LEEP. Of these, 105 women consented to participate and underwent preoperative ophthalmologic evaluation. Thirty women were excluded from the final analysis because of HPV negativity. The patient selection process is shown in Figure 1.

Normal ocular findings were observed in 66 women (88.0%), conjunctival/eyelid papilloma lesions in 3 women (4.0%), and other ocular findings in 6 women (8.0%). Among continuous variables, the median age was 40 (24–67) years. Median gravidity was 2 (0–9) and median parity was 2 (0–7). Median age at first intercourse was 21 (17–32) years.

Age, gravidity, parity, and age at first intercourse did not differ significantly between the three-category distribution of ocular findings groups. Alcohol use was reported in 6 of 62 patients with normal ocular findings, 2 of 3 patients with conjunctival/eyelid papilloma lesions, and none of the patients with other ocular findings; and differed across ocular finding groups (*p* = 0.039). No significant differences were observed in education, contraception use, smoking, or chronic disease history (all *p* > 0.05) (Table 1). Educational data were available for 54 women. Among these, high school was the most common educational level (22/54, 40.7%), followed by primary school (9/54, 16.7%) and university (9/54, 16.7%), middle school (5/54, 9.3%), and master’s degree (2/54, 3.7%).

### 3.2. Clinical and Ocular Findings According to Cervical Histopathology and HPV Subtype

The three-category distribution of ocular findings differed significantly according to colposcopic biopsy histopathology (*p* = 0.016), but not according to final LEEP histopathology (*p* = 0.465) or HPV subtype (*p* = 1.000). Among women with non-malignant/normal colposcopic biopsy findings, 3 of 5 (60.0%) had normal ocular findings and 2 (40.0%) had conjunctival/eyelid papilloma. In the CIN1 group, 13 of 16 (81.2%) had normal ocular findings, and 3 (18.8%) had other ocular findings. In the CIN2+ group, 50 of 54 (92.6%) had normal ocular findings, 1 (1.9%) had papilloma, and 3 (5.6%) had other ocular findings. Thus, papilloma was proportionally most frequent in the non-malignant/normal colposcopic category, whereas normal ocular findings predominated in CIN2+ lesions (Table 2).

By contrast, ocular findings did not differ significantly according to final LEEP histopathology. Normal ocular findings predominated in all LEEP categories, including non-malignant/normal (22/28, 78.6%), CIN1 (12/12, 100%), CIN2+ (26/29, 89.7%), and invasive cancer (3/3, 100%). Ocular findings also did not vary according to HPV subtype. Among women with HPV16/18, 25 of 28 (89.3%) had normal ocular findings; among women with non-16/18 HPV, 35 of 41 (85.4%) had normal ocular findings; and among women with HPV16/18 plus other types, all 6 had normal ocular findings.

### 3.3. Variables According to Any Ocular Pathology

Women with any ocular pathology were older than those with normal ocular findings (48.53 ± 9.21 vs. 39.27 ± 9.51 years, *p* = 0.002). Gravidity (3.67 ± 1.99 vs. 2.71 ± 1.98; *p* = 0.101), parity (2.73 ± 1.79 vs. 1.95 ± 1.45; *p* = 0.128), and age at first intercourse (23.87 ± 9.03 vs. 21.08 ± 3.11; *p* = 0.257) did not differ significantly in the main Welch *t*-test analysis. In the sensitivity Wilcoxon analysis, the age difference remained significant (*p* = 0.001), gravidity showed a weak difference (*p* = 0.046), parity remained non-significant (*p* = 0.068), and age at first intercourse remained non-significant (*p* = 0.360) (Table 3).

### 3.4. Exploratory Adjusted Analyses of Papilloma and Any Ocular Pathology

Exploratory Firth penalized logistic regression analyses were conducted because ocular pathology events were rare. In the age-only model for conjunctival/eyelid papilloma, age was not significantly associated with papilloma occurrence (OR 1.03, 95% CI 0.90–1.15; *p* = 0.683). In the sensitivity model that included age and dichotomized colposcopic biopsy findings, neither age nor CIN2+ colposcopic biopsy was significantly associated with conjunctival/eyelid papilloma.

For any ocular pathology, age was not significantly associated with the outcome after adjustment for colposcopic biopsy findings. Colposcopic CIN2+ showed lower odds of any ocular pathology than non-CIN2+ colposcopic findings, but this result was not statistically significant (OR 0.29, 95% CI 0.07–1.14; *p* = 0.075). In the LEEP sensitivity model, LEEP CIN2+/cancer was not significantly associated with any ocular pathology. Although alcohol use was associated with ocular finding groups in categorical comparisons, it was not statistically significant in the exploratory Firth model adjusted for age (OR 3.90, 95% CI 0.60–21.8; *p* = 0.144) (Table 4).

## 4. Discussion

In this prospective, cross-sectional study of women undergoing a loop electrosurgical excision procedure (LEEP) for cervical dysplasia, ocular abnormalities were observed in 12.0% (*n* = 9) of women in the final analytical cohort. The three-category distribution of ocular findings differed according to colposcopic biopsy histopathology (*p* = 0.016) but not with final LEEP histopathology (*p* = 0.465) or HPV subtype. Overall, ocular findings were uncommon, and conjunctival/eyelid papilloma was identified in only three patients. In exploratory adjusted analyses, no independent predictor of conjunctival/eyelid papilloma or any ocular pathology was identified.

Although cervical HPV positivity indicates the presence of the virus in the lower genital tract, ocular surface lesions develop in a different epithelial environment and are likely exposed to different local cofactors than those involved in cervical disease, and the broader HPV literature supports this view [7]. While HPV is central to cervical carcinogenesis and forms the basis of current screening and risk-based treatment strategies [4,21], HPV-related diseases observed in mucosal regions are not uniform in terms of their biological characteristics or clinical manifestations [7,22]. The oropharynx is the best-defined non-cervical model in which HPV-induced malignant transformation is well established [22]. However, even here, the interaction between viral status and local tissue context exhibits region-specific characteristics. Accordingly, the ocular surface should not be regarded as a direct extension of the cervical model [15,23].

The literature on ocular HPV is lesion-specific, and the strongest association has been reported in conjunctival papillomas [15,16,24,25]. Recent reviews and meta-analyses have supported this lesion-specific pattern, with the strongest evidence reported for conjunctival papilloma and ocular surface squamous neoplasia rather than across all ocular surface lesions [15,19,20]. Sjo et al. reported detecting HPV in 86 of 106 conjunctival papillomas, whereas all normal conjunctival samples were HPV-negative; HPV6 and HPV11 were the most prevalent types [16]. Reviews on conjunctival papillomas similarly conclude that low-risk HPV, particularly types 6 and 11, plays a significant role in a substantial subset of these lesions [24]. In contrast, evidence for other ocular surface lesions is inconsistent [15,19,23,26]. Reviews of ocular HPV disease and infections emphasize that HPV’s role in pterygium [27,28,29], and ocular surface squamous neoplasia is unclear and likely modified by ultraviolet exposure, immune status, and geographic location [15,19,23,26].

The existing literature on the cervical–ocular relationship is limited and does not support a strong epidemiological association. McDonnell et al. showed that HPV 16/18 DNA could be detected in ocular swabs from women with genital tract condylomata or cervical dysplasia even in the absence of clinically evident ocular lesions, suggesting that ocular HPV positivity may coexist with cervical HPV-related disease without necessarily indicating ocular pathology [30]. Similarly, a published case report describing concurrent cervical and ocular squamous lesions suggests a biological possibility; however, these are rare cases and cannot be generalized as evidence of a cervical–ocular pathway [31]. Recent reviews of ocular adnexal neoplasia indicate that HPV may play a role in selected conjunctival neoplasms, although the evidence remains heterogeneous beyond conjunctival papilloma [17,19,23]. The present findings are consistent with this limited and heterogeneous literature, as ocular abnormalities were infrequent and no robust independent association with cervical disease characteristics was demonstrated in exploratory adjusted analyses.

Ocular findings were associated with colposcopic biopsy histopathology, whereas no association was observed with final LEEP histopathology. This difference may result from the different nature of biopsy and excision specimens, including variations in sampling, lesion representation, and final histopathologic classification. Ocular abnormalities were more frequent in women with non-malignant or normal colposcopic biopsy findings than in those with CIN1 or CIN2+. Therefore, the distribution of ocular lesions did not increase with the severity of cervical disease in this cohort. Although women with ocular pathology were older in the initial comparison analysis, age was not significantly associated with ocular pathology in exploratory adjusted models.

### Strengths and Limitations

This study has several strengths. It was conducted prospectively in a clinically well-defined cohort of women scheduled for LEEP surgery due to cervical dysplasia, and an ophthalmological examination was included in the preoperative evaluation. This design allowed for the systematic assessment of ocular findings in a real-world gynecologic oncology clinic. The study also has limitations. It was conducted at a single center, and the number of ocular lesions was small. In particular, the very small number of papilloma cases limited the precision of the exploratory adjusted analyses. Ocular HPV genotyping was not performed; therefore, cervical HPV findings could not be directly compared with HPV status in ocular tissue. Furthermore, the ocular abnormalities examined in this study exhibited clinical heterogeneity, and the evidence correlating specific lesion types with HPV is not consistent. In addition, clinical images of ocular findings were not available for inclusion in the manuscript. Finally, because this was a cross-sectional study, it was not possible to determine whether the ocular findings occurred before, followed, or coincided with the cervical findings, and no causal relationship could be established.

## 5. Conclusions

In this prospective cohort of women with cervical HPV positivity undergoing LEEP for cervical dysplasia, most patients had normal ocular findings, and conjunctival/eyelid papilloma was rare. The findings do not support a clinically meaningful cervical–ocular association or routine ophthalmological screening based on cervical HPV positivity. Ocular evaluation should be guided by clinical ocular findings. Further studies with ocular tissue-based HPV testing are needed to investigate whether HPV is present in ocular lesions and whether these findings correspond to cervical HPV results. Therefore, the main contribution of this study is to raise awareness rather than to expand screening procedures.

## Figures and Tables

**Table 1:** Baseline clinicopathological characteristics of the study cohort according to ocular finding groups.

A. Continuous variables according to ocular finding groups *							
**Variable**			**Normal ocular findings**	**Conjunctival/eyelid papilloma**	**Other ocular findings**	**Test method**	***p*** **-value**
Age, years			39 (24–67)	39 (33–51)	44.5 (35–50)	Kruskal–Wallis	0.182
Gravida			2 (0–9)	2 (1–4)	3 (0–6)		0.87
Parity			2 (0–7)	1 (0–3)	2 (0–3)		0.793
Age at first intercourse, years			21 (17–28)	22 (20–24)	22.5 (17–32)		0.432
B. Categorical variables according to ocular finding groups **							
**Variable**	**Category**	**Total**	**Normal ocular findings**	**Conjunctival/eyelid papilloma**	**Other ocular findings**	**Test method**	***p*** **-value**
Contraception use	No	42 (60.0%)	37 (60.7%)	1 (33.3%)	4 (66.7%)	Fisher’s exact with simulation	0.734
	Yes	28 (40.0%)	24 (39.3%)	2 (66.7%)	2 (33.3%)		
Smoking	No	48 (66.7%)	43 (68.3%)	1 (33.3%)	4 (66.7%)		0.526
	Yes	24 (33.3%)	20 (31.7%)	2 (66.7%)	2 (33.3%)		
Alcohol use	No	62 (88.6%)	56 (90.3%)	1 (33.3%)	5 (100.0%)		0.039
	Yes	8 (11.4%)	6 (9.7%)	2 (66.7%)	0 (0.0%)		
Chronic disease	No	52 (72.2%)	47 (74.6%)	2 (66.7%)	3 (50.0%)		0.465
	Yes	20 (27.8%)	16 (25.4%)	1 (33.3%)	3 (50.0%)		
HPV subtype	HPV16/18	28 (37.3%)	25 (37.9%)	1 (33.3%)	2 (33.3%)		0.96
	Non-16/18	41 (54.7%)	35 (53.0%)	2 (66.7%)	4 (66.7%)		
	HPV16/18 + other	6 (8.0%)	6 (9.1%)	0 (0.0%)	0 (0.0%)		

* Data are shown as median (minimum–maximum). Continuous variables were compared with the Kruskal–Wallis test. Missing data were not imputed; analyses used available cases. The baseline variable set and the available-case approach align with the study definitions in the manuscript. ** Data are shown as n (%). Percentages were calculated based on available cases for each variable; therefore, denominators differ across variables because missing data were not imputed. Categorical variables were analyzed using Pearson’s chi-square test or Fisher’s exact test, as appropriate.

**Table 2:** Distribution of ocular findings according to colposcopic cervical histopathology.

Colposcopic Biopsy Category	Normal Ocular Findings	Conjunctival/Eyelid Papilloma	Other Ocular Findings	Test Method	*p* Value
Non -malignant/normal	3 (60.0%)	2 (40.0%)	0 (0.0%)	Fisher’s exact with simulation	0.016
CIN1	13 (81.2%)	0 (0.0%)	3 (18.8%)		
CIN2+	50 (92.6%)	1 (1.9%)	3 (5.6%)		

Data are presented as n (%). Ocular findings were classified as normal, conjunctival/eyelid papilloma lesions, or other ocular findings. Other ocular findings included pterygium, pinguecula, and benign eyelid masses. Due to sparse cell counts, categorical comparisons were performed using Fisher’s exact test with simulation for larger tables.

**Table 3:** Main analysis of continuous variables according to any ocular pathology status.

Variable	No Ocular Pathology	Ocular Pathology Present	Test Method	*p* Value
Age, years	39.27 ± 9.51	48.53 ± 9.21	Welch *t*-test	0.002
Gravida	2.71 ± 1.98	3.67 ± 1.99		0.101
Parity	1.95 ± 1.45	2.73 ± 1.79		0.128
Age at first intercourse, years	21.08 ± 3.11	23.87 ± 9.03		0.257

Data values are presented as mean ± standard deviation. “Any ocular pathology” was defined as conjunctival/eyelid papilloma or other ocular findings, with normal ocular findings as the reference category. Continuous variables were compared using Welch’s *t*-test.

**Table 4:** Exploratory Firth penalized logistic regression analyses for ocular findings.

Outcome/Model	Variable	Adjusted OR	95% CI	*p* Value
Papilloma vs. non-papilloma, age-only model	Age, per 1-year increase	1.03	0.90–1.15	0.683
Papilloma vs. non-papilloma, sensitivity model	Age, per 1-year increase	1.01	0.88–1.15	0.862
	Colposcopy CIN2+ vs. non-CIN2+	0.23	0.02–1.87	0.166
Any ocular pathology vs. normal, main model	Age, per 1-year increase	1.05	0.97–1.13	0.269
	Colposcopy CIN2+ vs. non-CIN2+	0.29	0.07–1.14	0.075
Any ocular pathology vs. normal, LEEP sensitivity model	Age, per 1-year increase	1.05	0.98–1.14	0.177
	LEEP CIN2+/cancer vs. non-CIN2+	0.63	0.14–2.47	0.516
Any ocular pathology vs. normal, alcohol sensitivity model	Age, per 1-year increase	1.05	0.97–1.14	0.257
	Alcohol use, yes vs. no	3.90	0.60–21.8	0.144

Adjusted odds ratios (ORs), 95% confidence intervals (CIs), and two-sided *p* values are presented from Firth penalized logistic regression models. Firth regression was chosen because papilloma events were rare.

## Data Availability

The data that support the findings of this study are available upon request from the corresponding author.

## Abbreviations

The following abbreviations are used in this manuscript:

LEEP	Loop electrosurgical excision procedure
HPV	Human Papillomavirus
CIN	Cervical intraepithelial neoplasia
